# Supplementary material for: Assessment of Childhood Stunting Prevalence over Time and Risk Factors of Stunting in the Healthy Village Programme Areas in Bangladesh
Source: Children (Basel). 2024 May 28;11(6):650. doi: 10.3390/children11060650 (PMC11202057; doi:10.3390/children11060650)
Supplement: Supplementary file 1 [file children-11-00650-s001.zip › children-2979782-supplementary.pdf]

children

MDPI

Supplementary Materials

File S1. Information on data sets and descriptive statistics of nutrition survey data

Table S1. Data sets used in the study of childhood stunting in programme areas in five districts (Barguna, Patuakhali, Jessore, Khulna, and Satkhira) of southern coastal Bangladesh.

Data set	Data collection (year)	Sample size (n)
Household census	2018	283 844
Anthropometric measurements (z-scores)	Oct 2018 to Dec 2021	563 323
Nutrition and child feeding practices	2018	864

Table S2. Types of drinks children received in the first three days of birth (n = 864).

Description	Freq.	Percent of cases
breastmilk only	661	77
drinks other than breastmilk	203	23
Total	864	100

Table S3. Types of drinks children received in the first six months of birth (n = 864).

Description	Freq.	Percent of cases
breastmilk only	445	52
drinks other than breastmilk	419	48
Total	864	100

Table S4. Type of micronutrient supplementation received by infants and young children (6–59 months) in households (n = 864).

Nutrient supplmentation types	Frequency	Percent of responses	Percent of cases
vitamin A supplementation	466	39	54
continued breastfeeding	330	27	38
complementary feeding	343	29	40
daily iron supplementation	51	4	6
others	11	1	1
Total	1201	100	139

Table S5. Child feeding frequency and quantity (12–24 months) (n = 864).

Description	Freq.	Percent of responses	Percent of cases
included animal-based foods (e.g., fish, eggs, chicken, liver, milk)	619	28	72
included a variety of orange and red vegetables and fruits	616	27	71
fed at least 3–4 times a day	602	27	70
fed family foods plus one or two snacks/solid foods	276	12	32
continued breastfeeding up to 24 months and beyond	111	5	13
used iodised salt to prepare food	21	1	2
Total	2 245	100	260

File S2: Further results

Table S6. Childhood stunting prevalence in programme areas in five districts of southern coastal Bangladesh from 2018 to 2021.

District	2018 (n= 26 707) Count (%)	2019 (n= 37 662) Count (%)	2020 (n= 34 236) Count (%)	2021 (n= 33 433) Count (%)	Percent reduction from 2018 to 2021
Barguna	354 (49%)	1 538 (64%)	1 170 (49%)	724 (27%)	46%
Jessore	1 108 (45%)	1 017 (46%)	338 (17%)	470 (28%)	38%
Khulna	2 714 (46%)	4 222 (49%)	1 544 (20%)	1 169 (14%)	69%
Patuakhali	6 061 (55%)	7 876 (49%)	5 971 (42%)	4 387 (30%)	46%
Satkhira	3 425 (51%)	5 374 (63%)	3 017 (37%)	1 447 (24%)	54%

The prevalence estimates were point-prevalence estimates, using Quarter 4 (October to December) data from 2018 to 2021.

Table S7. Univariate logistic regression analysis of risk factors for childhood stunting in programme areas in five districts of southern coastal Bangladesh (n=20 174).

Variables	Odds ratio	Standard error	z	P>|z|	95% CI	
**Child age group**	**0 to 1 year**					**Ref**
1 to 2 y	1.29	0.06	5.54	0.001***	1.18	1.43
2 to 3 y	0.99	0.05	-0.04	0.965	0.91	1.09
3 to 4 y	1.00	0.05	0.14	0.889	0.92	1.11
4 to 5 y †	0.92	0.05	-1.44	0.149	0.84	1.03
**Child gender**	**Female**					**Ref**
Male	1.09	0.03	3.02	0.001***	1.03	1.15
**Area type**	**New area**					**Ref**
Follow up	1.31	0.04	8.63	0.001***	1.23	1.40
**Mother's age group**	**<21**					**Ref**
21-30	0.99	0.04	-0.25	0.800	0.91	1.08
31-40	0.96	0.05	-0.75	0.450	0.87	1.07
>40	1.06	0.07	0.91	0.360	0.93	1.21
**Mother's education status**	**Higher education**					**Ref**
Illiterate	1.52	0.10	6.19	0.001***	1.33	1.73
Read and Write	1.65	0.12	7.08	0.001***	1.44	1.90
Primary	1.44	0.08	6.88	0.001***	1.30	1.59
Secondary	1.32	0.07	5.30	0.001***	1.19	1.46
**Household size**	**size <5**					**Ref**
size 5 to 7	1.01	0.03	0.41	0.680	0.95	1.07
size >7	0.96	0.05	-0.89	0.370	0.87	1.05
**Number of under-five children in a household**	**One under-five child**					**Ref**
More than one under-five child	1.15	0.05	3.39	0.001***	1.06	1.24

Variables	Odds ratio	Standard error	z	P>|z|	95% CI	
**Household socioeconomic status**	**Rich**					**Ref**
Hardcore poor	1.66	0.11	7.53	0.001***	1.46	1.90
Poor	1.65	0.10	8.32	0.001***	1.47	1.86
Middle-income	1.41	0.09	5.56	0.001***	1.25	1.59
**Household access to safe water for drinking**	**Yes**					**Ref**
No	0.51	0.18	-1.93	0.050*	0.26	1.01
**Household access to safe water for cooking**	**Yes**					**Ref**
No	1.25	0.04	7.68	0.001***	1.18	1.32
**Household access to safe water for washing utensils**	**Yes**					**Ref**
No	1.11	0.03	3.17	0.001***	1.04	1.18
**Household access to safe water for washing children's clothes**	**Yes**					**Ref**
No	1.00	0.04	-0.07	0.940	0.93	1.07
**Water source**	**Deep tubewell**					**Ref**
Others (shallow tubewell, treated pond water, etc.)	0.89	0.03	-4.12	0.001***	0.84	0.94
**Household access to water source all year round†**	**Yes**					**Ref**
No	0.92	0.05	-1.61	0.110	0.83	1.02
**Self-investment in water sources**	**Yes**					**Ref**
No	1.11	0.03	3.46	0.001***	1.05	1.18
**Water source ownership**	**Self-owned**					**Ref**
Shared or owned by others	1.23	0.05	5.77	0.001***	1.15	1.33
**Distance from household to water source**	**<30 mins**					**Ref**
>30 mins or no access at all	1.02	0.03	0.62	0.532	0.96	1.09
**Household access to latrines**	**Yes**					**Ref**
No	0.97	0.14	-0.22	0.830	0.73	1.28
**Type of latrine**	**Improved pit latrine**					**Ref**
Unimproved pit latrine/hanging/open defecation	1.20	0.03	6.43	0.001***	1.13	1.27
**Latrine ownership**	**Self-owned**					**Ref**

Variables	Odds ratio	Standard error	z	P>|z|	95% CI	
Shared	0.98	0.04	-0.52	0.600	0.91	1.06
**Self-investment in latrines**	**Yes**					**Ref**
No	0.98	0.04	-0.42	0.670	0.90	1.07
**Distance from household to latrine**	**Attached or within 12 steps**					**Ref**
More than 12 steps or no access at all	1.03	0.03	1.04	0.300	0.97	1.09
**Monitoring of child growth by households**	**Yes**					**Ref**
No	1.30	0.07	4.92	0.001***	1.17	1.44

* p<0.05, ** p<0.01, *** p<0.001. 95% CI, 95% confidence interval. † Variables with p values >0.05 but < 0.25 that were included for multivariate analyses.

Table S8. Generalised linear regression analysis of risk factors for childhood stunting in programme areas in five districts of southern coastal Bangladesh (n = 20 174).

	Cluster by village (n = 20 174)	Cluster by union (n = 20 174)	Cluster by district (n = 20 174)
Covariates	OR (95% CI)	OR (95% CI)	OR (95% CI)
**Child age group**			
0 - 1 y	1.00 [1.00,1.00]	1.00 [1.00,1.00]	1.00 [1.00,1.00]
1 to 2 y	1.31*** [1.18,1.46]	1.32*** [1.16,1.50]	1.31*** [1.18,1.46]
2 to 3 y	1.00 [0.89,1.12]	1.00 [0.87,1.15]	1.00 [0.82,1.21]
3 to 4 y	1.00 [0.89,1.13]	1.00 [0.84,1.20]	1.00 [0.75,1.34]
4 to 5 y	0.94 [0.82,1.06]	0.94 [0.78,1.13]	0.94 [0.70,1.25]
**Child gender**			
Female	1.00 [1.00,1.00]	1.00 [1.00,1.00]	1.00 [1.00,1.00]
Male	1.10** [1.04,1.17]	1.10** [1.04,1.17]	1.10*** [1.06,1.14]
**Programme areas**			
New area	1.00 [1.00,1.00]	1.00 [1.00,1.00]	1.00 [1.00,1.00]
Follow up	1.24** [1.08,1.42]	1.18 [0.92,1.51]	1.23** [1.06,1.42]
**Mother's education status**			
Higher	1.00 [1.00,1.00]	1.00 [1.00,1.00]	1.00 [1.00,1.00]
Illiterate	1.27** [1.09,1.47]	1.26* [1.04,1.53]	1.28***[1.20,1.36]
Read and Write	1.37*** [1.16,1.62]	1.35** [1.12,1.64]	1.37** [1.09,1.73]
Primary	1.22** [1.07,1.38]	1.20* [1.04,1.38]	1.22*** [1.11,1.33]
Secondary	1.21** [1.08,1.36]	1.20** [1.07,1.36]	1.21*** [1.11,1.33]
**Number of under-five children in a household**			
One under-five child	1.00 [1.00,1.00]	1.00 [1.00,1.00]	1.00 [1.00,1.00]

	Cluster by village (n = 20 174)	Cluster by union (n = 20 174)	Cluster by district (n = 20 174)
Covariates	OR (95% CI)	OR (95% CI)	OR (95% CI)
More than one under-five child	1.14** [1.04,1.25]	1.14*** [1.05,1.23]	1.16***[1.09,1.24]
**Household socioeconomic status**			
Rich	1.00 [1.00,1.00]	1.00 [1.00,1.00]	1.00 [1.00,1.00]
Hardcore poor	1.44*** [1.22,1.71]	1.47** [1.13,1.92]	1.43* [1.06,1.92]
Poor	1.49*** [1.29,1.73]	1.51*** [1.22,1.86]	1.48***[1.21,1.80]
Middle-income	1.32*** [1.15,1.52]	1.32** [1.08,1.62]	1.31* [1.03,1.67]
**Self-investment in water sources**			
Yes	1.00 [1.00,1.00]	1.00 [1.00,1.00]	1.00 [1.00,1.00]
No	0.97 [0.88,1.08]	0.98 [0.86,1.11]	0.98 [0.85,1.12]
**Water ownership**			
Self-owned	1.00 [1.00,1.00]	1.00 [1.00,1.00]	1.00 [1.00,1.00]
Shared or owned by others	1.11 [0.98,1.26]	1.10 [0.92,1.32]	1.11 [0.95,1.29]
**Type of latrine**			
Improved pit latrine	1.00 [1.00,1.00]	1.00 [1.00,1.00]	1.00 [1.00,1.00]
Unimproved pit latrine/hanging/open defecation	1.10* [1.02,1.19]	1.09 [1.00,1.20]	1.10* [1.02,1.19]
**Monitoring of child growth by household**			
Yes	1.00 [1.00,1.00]	1.00 [1.00,1.00]	1.00 [1.00,1.00]
No	1.25** [1.06,1.48]	1.23 [0.95,1.60]	1.25* [1.01,1.55]
**Water source used by household**			
Tubewell (Deep)	1.00 [1.00,1.00]	1.00 [1.00,1.00]	1.00 [1.00,1.00]
Others (shallow tubewell, treated pond)	1.03 [0.92,1.16]	1.03 [0.84,1.27]	1.03 [0.83,1.28]

* p<0.05, ** p<0.01, *** p<0.001. OR, odds ratio; 95% CI, 95% confidence interval. For cluster analysis by village, union, and district, a multilevel generalised linear regression analysis using the binomial family and logit link was performed.
